# Network pharmacology-based analysis in determining the mechanisms of Huoxin pill in protecting against myocardial infarction

**DOI:** 10.1080/13880209.2021.1964542

**Published:** 2021-09-07

**Authors:** Jia He, Da Wo, En Ma, Qing Wang, Jinxiao Chen, Jun Peng, Weidong Zhu, Dan-ni Ren

**Affiliations:** a Fujian Key Laboratory of Integrative Medicine on Geriatric, Academy of Integrative Medicine, Fujian University of Traditional Chinese Medicine, Fuzhou, China; b Clinical and Translational Research Center, Research Institute of Heart Failure, Shanghai East Hospital, Key Laboratory of Arrhythmias of Ministry of Education, Tongji University School of Medicine, Shanghai, China

**Keywords:** Myocardial ischaemia, cardiovascular disease, bioactive compounds, pharmacological analysis, inflammatory response

## Abstract

**Context:**

Huoxin pill (HXP) is a commonly used TCM prescription for treatment of cardiovascular diseases. However, its mechanism in protecting against myocardial infarction (MI) remains unknown.

**Objective:**

We performed a network pharmacology analysis to explore the bioactive ingredients, therapeutic effects, and mechanisms of HXP in protecting against MI.

**Materials and methods:**

HPLC was used to identify major bioactive compounds, and overlap with MI target genes were visualised. 10-Week old C57BL/6 mice were randomly assigned as: Sham-operated control, MI + Phosphate buffered saline (PBS), and MI + HXP (3 mg/mL and 9 mg/mL) treatment groups, received oral gavage administration once every two-days starting from 1-week prior to MI, and subsequently MI models were established for one-week before sacrifice.

**Results:**

AKT1, VEGFA, TNF and RELA were identified as core target proteins among eighty-five candidate bioactive compounds identified in HXP with overlapping MI-related genes. HXP protection against MI was mainly via regulation of inflammatory pathways, notably TNF signalling pathway. Mouse models of MI and cardiac myoblasts demonstrated that HXP improved MI-induced injury via improving regulation of inflammatory response.

**Discussion and conclusion:**

Stellasterol, deoxycholic acid, kaempferol, and quercetin are important active compounds contained in HXP with anti-inflammatory properties in the therapeutic treatment of MI. Due to the straightforward nature and effectiveness of taking oral HXP medications, our findings provide a theoretical basis for the clinical application of HXP in treating patients with angina or myocardial ischaemia. Future research into the combination of surgical procedures or medications that restore blood flow together with HXP as supportive medication would be worthwhile.

## Introduction

Myocardial infarction (MI) is one of the leading causes of morbidity and mortality worldwide, triggering irreversible myocardial cell damage and heart failure. MI causes an intense inflammatory response that is critical for cardiac repair, but excess or prolonged activation of inflammatory cytokines can often result in worsened cardiac function, leading to post-infarction remodelling and heart failure (Frangogiannis 2014). Therefore, the timely suppression of the inflammatory response is essential to allow the beneficial recruitment of inflammatory factors to take place without compromising the overall response.

Network pharmacology has become one of the most important methods used in systems biology (Colinge et al. 2012), and has shown promising results in understanding the underlying therapeutic mechanisms of TCM prescriptions (Zhao et al. 2019). Machine-learning algorithms that can predict oral bioavailability, drug-likeness, Caco-2 permeability index and drug targets, can help the rapid identification of active TCM ingredients and classification of putative targets (Xue et al. 2013; Huang et al. 2014; Ru et al. 2014). Network spread-like algorithms can recognise protein-protein interactions within a network (Liu et al. 2009), while algorithms for finding hub nodes in networks facilitate the identification of putative targets (Liao et al. 2018).

Huoxin pill (HXP) is a commonly used TCM prescription clinically proven to be beneficial in the treatment of cardiovascular diseases, including coronary heart disease, MI and angina (Bo-Wen et al. 2018; Xu et al. 2019). HXP has been shown to improve microcirculation, increase coronary blood flow, enhance cardiac function, and improve myocardial contractility, with good clinical safety and tolerance (Liang et al. 2018). However, the mechanism of action of HXP in protecting against cardiovascular diseases, in particular myocardial ischaemia remains poorly understood. In this study, we utilised network pharmacology based analysis to select for the main potential active compounds and target genes involved in the therapeutic effect of HXP in protecting against MI, and subsequent experimental validation to verify the potential underlying mechanisms involved.

## Materials and methods

### Preparation of HXP Extract

Huoxin pill (Batch: 15050101) was provided by Guangzhou Youcare Biopharmaceutics Co., Ltd. (Guangzhou, China). The constituents of Huoxin pill consisted of: *Ganoderma lucidum* (Curtis) Kummer (Ganodermataceae), *Abelmoschus moschatus* Medicus (Malvaceae), *Ursi Fellis Pulvis* (*Selenarctos thibetanus* Cuvier) (Ursidae), *Bezoar bovis* (*Bos taurus domesticus* Gmelin) (Bovidae), *Pteria martensii* (Dunker) (Pteriidae), *Panax ginseng* C.A. Meyer (Araliaceae), *Bufonis Venenum* (*Bufo bufo gargarizans* Cantor) (Bufonidae), *Aconitum carmichaelii* (Debeaux) (Ranunculaceae), Borneolum syntheticum (Borneol) Camphor, *Carthamus tinctorius* L. (safflower) (Asteraceae), and were mixed at a ratio of 33:2:4:2:4:30:3:15:2:3. To prepare the extract, HXP was ground into a fine powder that passes through a sieve with nominal mesh aperture of 180 μm. The dried powder was re-dissolved in phosphate buffered saline (PBS) to a concentration of 10 mg/mL, filtered with a 0.22 μm filter and stored at −20 °C for further use.

### Component analysis of HXP powder using high-performance liquid chromatography-mass spectrometry (HPLC-MS/MS)

Fingerprinting analysis was conducted using HPLC-MS/MS to identify the chemical profile of top bioactive compounds in HXP powder extract (0.3 g) was accurately weighed and extracted with 5 mL HPLC grade methanol in an ultrasonic bath for 4 h. The extraction solution was diluted five times and filtered with a 0.22 μm microporous membrane for HPLC-MS/MS system (LC-30A, Shimadzu, Japan) and separated on a C18 ODS column (1.8 μm, 2.1 × 100 mm) with gradient elution. 2-Sulfobenzoic acid hydrate (0.2%) (A) and acetonitrile (B) were used as mobile phase and the gradient elution procedure was as follows: 0 min, A:B = 97:5; 0.01 min, A:B = 75:30; 37 min, A:B = 95:5; 37.1 min. The flow rate was 0.5 mL/min.

### Database construction and bioactive evaluation of compounds

We obtained all bioactive compounds identified in HXP (Tables 1 and 2) from the Traditional Chinese medicine system pharmacology technology platform (TCMSP, http://tcmspw.com/tcmsp.php) database and Traditional Chinese medicine integrated database (TCMID, http://www.megabionet.org/tcmid/). These databases contain comprehensive and up-to-date information of herbs, ingredients, and drug targets for drug screening and evaluation. According to the criteria suggested by the TCMSP, oral bioavailability (OB) ≥ 30%, drug-likeness (DL) ≥ 0.18, and Caco-2 permeability (Caco-2) ≥ −0.4 were selected parameters used to determine the bioactivity of compounds. The names of all compounds were standardised based on PubChem CIDs (https://pubchem.ncbi.nlm.nih.gov/), and listed according to whether the components were sourced from natural herbs (Table 1), or animal or synthetic sources (Table 2). We further used SwissTarget prediction tool, which compares 2D and 3D similarity measures with known ligands, in order to predict the potential targets of HXP bioactive compounds (Gfeller et al. 2014).

**Table 1.** Potential active compounds sourced from natural herbs in HXP.

Pubchem CID	Molecule Name	Herb	Database	
14137634	Ganoderic acid Mf	*Ganoderma lucidum* (Curtis) Kummer	TCMSP	
91820274	Ganolucidate A	*Ganoderma lucidum* (Curtis) Kummer	TCMSP	
21633085	Methyl lucidenate F	*Ganoderma lucidum* (Curtis) Kummer	TCMSP	
5283669	Stellasterol	*Ganoderma lucidum* (Curtis) Kummer	TCMSP	
14015434	Epoxyganoderiol A	*Ganoderma lucidum* (Curtis) Kummer	TCMSP	
14015436	Epoxyganoderiol B	*Ganoderma lucidum* (Curtis) Kummer	TCMSP	
14015438	Epoxyganoderiol C	*Ganoderma lucidum* (Curtis) Kummer	TCMSP	
56676695	CHEMBL1801892	*Ganoderma lucidum* (Curtis) Kummer	TCMSP	
11177299	Ganodesterone	*Ganoderma lucidum* (Curtis) Kummer	TCMSP	
139585889	Ergosta palmitate	*Ganoderma lucidum* (Curtis) Kummer	TCMSP	
101449382	Ergosta pentadecanoate	*Ganoderma lucidum* (Curtis) Kummer	TCMSP	
5351516	Peroxyergosterol	*Ganoderma lucidum* (Curtis) Kummer	TCMSP	
14015440	Ganoderal B	*Ganoderma lucidum* (Curtis) Kummer	TCMSP	
10097521	Ganoderic acid beta	*Ganoderma lucidum* (Curtis) Kummer	TCMSP	
11784642	Ganoderic acid DM	*Ganoderma lucidum* (Curtis) Kummer	TCMSP	
131751712	Ganoderic acid Mi	*Ganoderma lucidum* (Curtis) Kummer	TCMSP	
11442745	Ganoderic acid TR	*Ganoderma lucidum* (Curtis) Kummer	TCMSP	
101600073	Ganoderic acid V	*Ganoderma lucidum* (Curtis) Kummer	TCMSP	
131752702	Ganoderic acid V1	*Ganoderma lucidum* (Curtis) Kummer	TCMSP	
101600075	Ganoderic acid X	*Ganoderma lucidum* (Curtis) Kummer	TCMSP	
57397445	Ganoderic acid Y	*Ganoderma lucidum* (Curtis) Kummer	TCMSP	
10601916	Ganoderic acid Z	*Ganoderma lucidum* (Curtis) Kummer	TCMSP	
14484704	Ganoderic aldehyde A	*Ganoderma lucidum* (Curtis) Kummer	TCMSP	
471008	Ganoderiol F	*Ganoderma lucidum* (Curtis) Kummer	TCMSP	
73294	Ganodermanondiol	*Ganoderma lucidum* (Curtis) Kummer	TCMSP	
21124247	Ganodermatriol	*Ganoderma lucidum* (Curtis) Kummer	TCMSP	
6439006	Ganodermenonol	*Ganoderma lucidum* (Curtis) Kummer	TCMSP	
9985134	Ganodermic acid R	*Ganoderma lucidum* (Curtis) Kummer	TCMSP	
10436380	Ganodermic acid T-Q	*Ganoderma lucidum* (Curtis) Kummer	TCMSP	
13934284	Ganoderol A	*Ganoderma lucidum* (Curtis) Kummer	TCMSP	
15602283	Ganolucidic acid E	*Ganoderma lucidum* (Curtis) Kummer	TCMSP	
11048424	Lucialdehyde A	*Ganoderma lucidum* (Curtis) Kummer	TCMSP	
10343868	Lucialdehyde B	*Ganoderma lucidum* (Curtis) Kummer	TCMSP	
10366713	Lucidal	*Ganoderma lucidum* (Curtis) Kummer	TCMSP	
71453988	Lucidone A	*Ganoderma lucidum* (Curtis) Kummer	TCMSP	
475410	Lucidumol A	*Ganoderma lucidum* (Curtis) Kummer	TCMSP	
11271456	Methyl lucidenate Q	*Ganoderma lucidum* (Curtis) Kummer	TCMSP	
10181133	Cerevisterol	*Ganoderma lucidum* (Curtis) Kummer	TCMSP	
222284	β-Sitosterol	*Ganoderma lucidum* (Curtis) Kummer	TCMSP	
33934	Diop	*Panax ginseng,* (C.A. Meyer)	TCMSP	
5280794	Stigmasterol	*Panax ginseng,* (C.A. Meyer)	TCMSP	
91510	Maackiain	*Panax ginseng,* (C.A. Meyer)	TCMSP	
5280863	Kaempferol	*Panax ginseng,* (C.A. Meyer)	TCMSP	
21160900	Chrysanthemaxanthin	*Panax ginseng,* (C.A. Meyer)	TCMSP	
442847	Celabenzine	*Panax ginseng,* (C.A. Meyer)	TCMSP	
285342	Deoxyharringtonine	*Panax ginseng,* (C.A. Meyer)	TCMSP	
441562	Dianthramine	*Panax ginseng,* (C.A. Meyer)	TCMSP	
444899	Arachidonic acid	*Panax ginseng,* (C.A. Meyer)	TCMSP	
441965	Frutinone A	*Panax ginseng,* (C.A. Meyer)	TCMSP	
21599928	Ginsenoside Rh4	*Panax ginseng,* (C.A. Meyer)	TCMSP	
96943	Girinimbin	*Panax ginseng,* (C.A. Meyer)	TCMSP	
6438572	Gomisin B	*Panax ginseng,* (C.A. Meyer)	TCMSP	
73498	Panaxadiol	*Panax ginseng,* (C.A. Meyer)	TCMSP	
132350840	Suchilactone	*Panax ginseng,* (C.A. Meyer)	TCMSP	
5742590	Sitogluside	*Panax ginseng,* (C.A. Meyer)	TCMSP	
11550001	Ginsenoside Rg5	*Panax ginseng,* (C.A. Meyer)	TCMSP	
4970	Protopine	*Panax ginseng,* (C.A. Meyer)	TCMSP	
5282805	Eicosadienoic acid	*Aconitum carmichaelii* (Debeaux)	TCMSP	
10100906	Delphin	*Aconitum carmichaelii* (Debeaux)	TCMSP	
906525	Deltoin	*Aconitum carmichaelii* (Debeaux)	TCMSP	
21679042	Deoxyandrographolide	*Aconitum carmichaelii* (Debeaux)	TCMSP	
441742	Karakoline	*Aconitum carmichaelii* (Debeaux)	TCMSP	
100633	Karanjin	*Aconitum carmichaelii* (Debeaux)	TCMSP	
138111911	Neokadsuranic acid B	*Aconitum carmichaelii* (Debeaux)	TCMSP	
3075701	Benzoylnapelline	*Aconitum carmichaelii* (Debeaux)	TCMSP	
21598997	Deoxyaconitine	*Aconitum carmichaelii* (Debeaux)	TCMSP	
440988	(*R*)-Norcoclaurine	*Aconitum carmichaelii* (Debeaux)	TCMSP	
3035320	Ignavine	*Aconitum carmichaelii* (Debeaux)	TCMSP	
16401028	Isotalatizidine	*Aconitum carmichaelii* (Debeaux)	TCMSP	
11953915	Carnosifloside I	*Aconitum carmichaelii* (Debeaux)	TCMSP	
441737	Hypaconitine	*Aconitum carmichaelii* (Debeaux)	TCMSP	
457801	Clionasterol	*Carthamus tinctorius* L. (safflower)	TCMSP	
5281238	Flavoxanthin	*Carthamus tinctorius* L. (safflower)	TCMSP	
261166	Lignan	*Carthamus tinctorius* L. (safflower)	TCMSP	
161739	Lupeol-palmitate	*Carthamus tinctorius* L. (safflower)	TCMSP	
5280784	Phytoene	*Carthamus tinctorius* L. (safflower)	TCMSP	
6436722	Phytofluene	*Carthamus tinctorius* L. (safflower)	TCMSP	
5281555	Pyrethrin II	*Carthamus tinctorius* L. (safflower)	TCMSP	
5281638	6-Hydroxykaempferol	*Carthamus tinctorius* L. (safflower)	TCMSP	
5281605	Baicalein	*Carthamus tinctorius* L. (safflower)	TCMSP	
5281241	Carthamone	*Carthamus tinctorius* L. (safflower)	TCMSP	
188308	Carthamidin	*Carthamus tinctorius* L. (safflower)	TCMSP	
5281680	Quercetagetin	*Carthamus tinctorius* L. (safflower)	TCMSP	
5280489	β-Carotene	*Carthamus tinctorius* L. (safflower)	TCMSP	
5280445	Luteolin	*Carthamus tinctorius* L. (safflower)	TCMSP	
5280343	Quercetin	*Carthamus tinctorius* L. (safflower)	TCMSP	
5997	Cholesterol	*Abelmoschus moschatus* (Medicus)	TCMID	
10947	Muscone	*Abelmoschus moschatus* (Medicus)	TCMID	
193306	Muscopyridine	*Abelmoschus moschatus* (Medicus)	TCMID	
13133503	Pentamethylenepyridine	*Abelmoschus moschatus* (Medicus)	TCMID	

**Table 2.** Potential active compounds sourced from animal or synthetic sources in HXP.

Pubchem CID	Molecule Name	Animal or Synthetic Compound	Database
119034	Asiatic acid	*Borneolum Syntheticum* (Borneol)	TCMSP
93009	Bronyl acetate	*Borneolum Syntheticum* (Borneol)	TCMSP
441676	Dipterocarpol	*Borneolum Syntheticum* (Borneol)	TCMSP
229346	Methyl desoxycholate	*Bezoar bovis* (Bos taurus domesticus Gmelin)	TCMSP
222528	Deoxycholic Acid	*Bezoar bovis* (Bos taurus domesticus Gmelin)	TCMSP
6917974	Resibufogenin	*Bufonis Venenum* (Bufo bufo gargarizans Cantor)	TCMID
222284	Sistosterol	*Bufonis Venenum* (Bufo bufo gargarizans Cantor)	TCMID
57030930	Methylcholesterol	*Bufonis Venenum* (Bufo bufo gargarizans Cantor)	TCMID
10092398	Epoxyresibufogenin	*Bufonis Venenum* (Bufo bufo gargarizans Cantor)	TCMID
10112	Calcium carbonate	*Pteria martensii* (Dunker)	TCMID
23925	Iron	*Pteria martensii* (Dunker)	TCMID
2758	Cineole	*Ursi Fellis Pulvis* (Selenarctos thibetanus Cuvier)	TCMID
6654	α-Pinene	*Ursi Fellis Pulvis* (Selenarctos thibetanus Cuvier)	TCMID
526762	α-Terpinen	*Ursi Fellis Pulvis* (Selenarctos thibetanus Cuvier)	TCMID
10133	Chenodeoxycholic acid	*Ursi Fellis Pulvis* (Selenarctos thibetanus Cuvier)	TCMID
221493	Cholic acid	*Ursi Fellis Pulvis* (Selenarctos thibetanus Cuvier)	TCMID
14896	β-Pinene	*Ursi Fellis Pulvis* (Selenarctos thibetanus Cuvier)	TCMID
387316	Taurochenodeoxycholic acid	*Ursi Fellis Pulvis* (Selenarctos thibetanus Cuvier)	TCMID
6675	Taurocholic acid	*Ursi Fellis Pulvis* (Selenarctos thibetanus Cuvier)	TCMID
11230	Terpinen	*Ursi Fellis Pulvis* (Selenarctos thibetanus Cuvier)	TCMID
222528	Deoxycholic acid	*Ursi Fellis Pulvis* (Selenarctos thibetanus Cuvier)	TCMID

### Collection of gene targets for MI

MI-related targets were identified from two existing resources: (1) We searched for the keywords ‘myocardial infarction’ in the DisGeNET database (http://www.disgenet.org/web/DisGeNET/menu) (Pinero et al. 2017) and obtained a total of 281 targets; (2) We searched for the keywords ‘myocardial infarction’ in Gene Cards Database (http://www.genecards.org/) (Safran et al. 2010) and obtained a total of 4643 known targets.

### Network construction of interactions between compounds and overlapping genes

The overlapping genes between compounds and MI target genes were identified and visualised using Venn diagrams and plotted using OmicShare platform (www.omicshare.com/tools). Based on the results of STRING database (https://string-db.org/), the PPI network for the targets of overlapping genes was obtained. Subsequently, the PPI network was analysed using Cytoscape (https://cytoscape.org/) based on the topological property of each node in the interaction network, namely: degree, betweenness centrality, and degree centrality.

### Functional annotation of key targets

The gene ontology (GO) and pathway enrichment analyses were conducted using the functional annotation tool of DAVID Bioinformatics Resources (http://david.abcc.ncifcrf.gov/). *p*-Value was calculated and further corrected using the Benjamin–Hochberg method. A false discovery rate < 0.05 was selected as the cut-off criterion. GO enrichment analysis and bubble chart of associated KEGG pathways were plotted using the OmicShare tools.

### Molecular docking

The crystal structures of candidate protein targets of HXP bioactive compounds were downloaded from the RCSB Protein Data Bank (http://www.pdb.org/) and modified using Autodock 4.2 software, including ligand and water removal, hydrogen addition, and amino acid optimisation and patching. ChemBioDraw 3D was used to create 3-dimensional chemical structures with the lowest energy. Results were saved in MOL.2 format. AutoDock Vina predicted docking partners by comparing the predicted conformation with the observed crystal structure. Binding energy of receptor and ligand lower than 0 kcal/mol represented a high affinity for docking (Sato et al. 2020).

### Animal studies

All animal studies were performed in accordance with institutional guidelines for the ethical care of laboratory animals and approved by the University Committee on the Care and Use of Laboratory Animals of Fujian University of Traditional Chinese Medicine (Approval NO.FJTCM IACUC 2019040).

C57BL/6 mice (10-12 weeks of age) were randomly assigned to four groups (*n* = 12 for each group): Sham, MI + PBS, MI + HXP (3 mg/kg.d) and 9 (mg/kg.d). Mice were anaesthetised by intraperitoneal injection of sodium pentobarbital (50 mg/kg) and all animal surgical procedures and subsequent analyses were performed by a blinded investigator. MI was performed by ligation of the proximal left anterior descending coronary artery (LAD). Sham mice received the same surgical procedure other than LAD ligation. HXP (9 mg/mL) or an equal volume of PBS was given via oral gavage once every two days for 1 week prior to and following MI.

### Two-dimensional echocardiography

Two-dimensional echocardiography was performed using a Vevo 2100 Imaging System (VisualSonics, Canada). Mice were anaesthetised using 1% isoflurane (Sigma Aldrich, St. Louis, MO, USA) supplemented with oxygen using a vaporiser (EZ Anaesthesia, Palmer, PA, USA). M-mode measurements were used to determine LV dimensions, including left ventricular internal dimension in diastole (LVID: d) and systole (LVID: s), which were respectively taken at the maximum ventricular size and the maximum contraction of the posterior wall. The LV ejection fraction (EF%) was calculated using the following equation: (LVIDd^3^ – LVIDs^3^)/LVIDd^3^ × 100. Fractional shortening (FS%) was calculated by using the following equation: (LVIDd – LVIDs)/LVIDd × 100. To minimise trauma associated with repeated anaesthesia, the mice did not undergo immediate assessment by echocardiography following MI operation.

### Real-time PCR assay

Total RNA was extracted using TRIzol reagent (Takara Biotechnology, China). Total RNA was reverse-transcribed to cDNA using PrimeScript II cDNA Synthesis Kit (Takara Biotechnology) according to the manufacturer’s instructions. Real-time quantitative PCR was performed with SYBR-Green master mix (Applied Biosystems, Foster City, CA, USA) in 96-well optical plates by using a QuantStudio 6 Flex Real-Time PCR System (Thermo Fisher Scientific). GAPDH was used as the reference gene for determination of relative gene expressions. Results are representative of at least three independent experiments.

### Cell culture and treatment

Adult rat myoblast H9c2 cells were used for *in vitro* experiments. Cells were seeded in 60 mm dishes using high-glucose Dulbecco’s modified Eagle’s medium (DMEM; Gibco, USA) and cultured at 37 °C in an incubator containing 5% CO_2_. To induce an inflammatory response, cells were pre-treated with 1.5 μg/mL HXP or PBS for 12 h, then treated with 10 ng/mL recombinant human TNF-alpha protein (TNF-α; R&D Systems, USA) for 4 h.

### Western blotting assay

Total protein was extracted by using RIPA buffer (Beyotime Biotechnology), and nuclear proteins were extracted using Nucleoprotein Extraction kit (#C500009, Sangon Biotech, China). Proteins were resolved on SDS-PAGE gels and transferred onto PVDF membranes, blocked with non-fat dry milk, and subsequently incubated with primary antibodies for (anti-IκBα, #9242, CST; anti-Phospho-IκBα, #2859, CST; anti-NF-κB1 p105/p50, #13586, CST; anti-NF-κB p65, #8242, CST; anti-TBP, #8515, CST; GAPDH, #5174, CST) overnight at 4 °C. Membranes were then incubated with the appropriate HRP-conjugated secondary antibodies for 1 h at room temperature and detected via chemiluminescence using Immobilon Chemiluminescent HRP Substrate (Millipore). Results are representative of at least two independent experiments.

### Statistical analysis

All statistical analyses were performed using Prism 5 software. Data were expressed as the mean ± standard error. *p* Values of <0.05 were considered statistically significant.

## Results

### Identification of potential bioactive compounds in HXP

**Figure 1.**
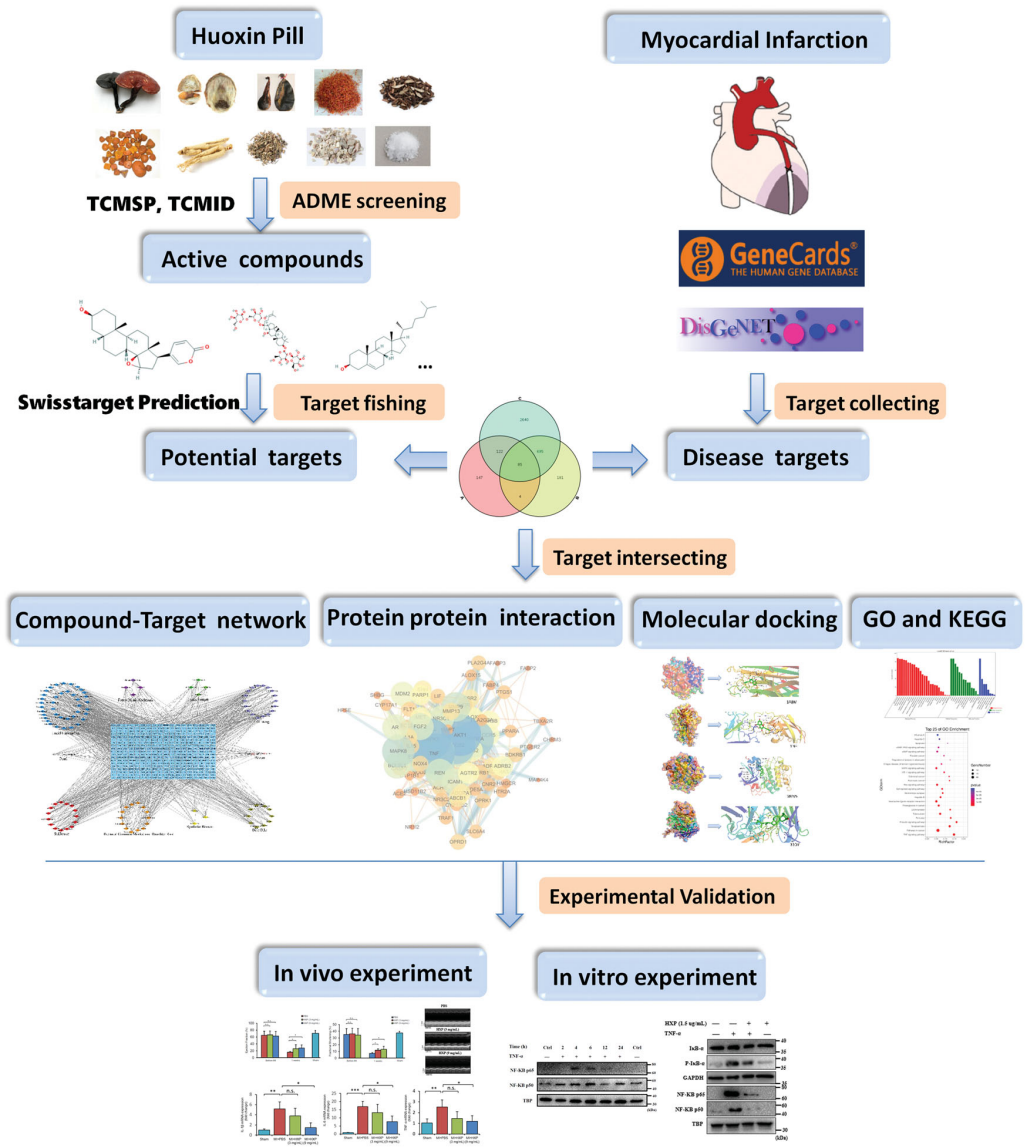
Integrated workflow for elucidating the mechanisms of HXP in the treatment of MI.

A detailed flow chart of our current study including pharmacology based analysis and experimental validation was shown in Figure 1. We first identified a total of 736 compounds from the HXP constituents: LG, GS, MR, SF, CB and SB were retrieved from the TCMSP database, and 20 compounds from the remaining HXP constituents: TV, MA, BB and pearl were obtained from TCMID database. Components that met the requirements of OB ≥30%, DL index ≥0.18 and Caco-2 threshold ≥−0.40 were selected, and after accounting for overlaps, a total of 111 components were chosen as candidate bioactive components for further analyses (Tables 1 and 2). Among these bioactive components, there were four high-degree components associated with multiple HXP targets, namely, stellasterol (PUBCHEM CID5283669, degree = 20), deoxycholic acid (PUBCHEM CID 222528, degree = 15), kaempferol (PUBCHEM CID 5280863, degree = 7), quercetin (PUBCHEM CID 5280343, degree = 7).

### High-performance liquid chromatography-mass spectrometry (HPLC-MS/MS) analysis of HXP powder

**Figure 2.**
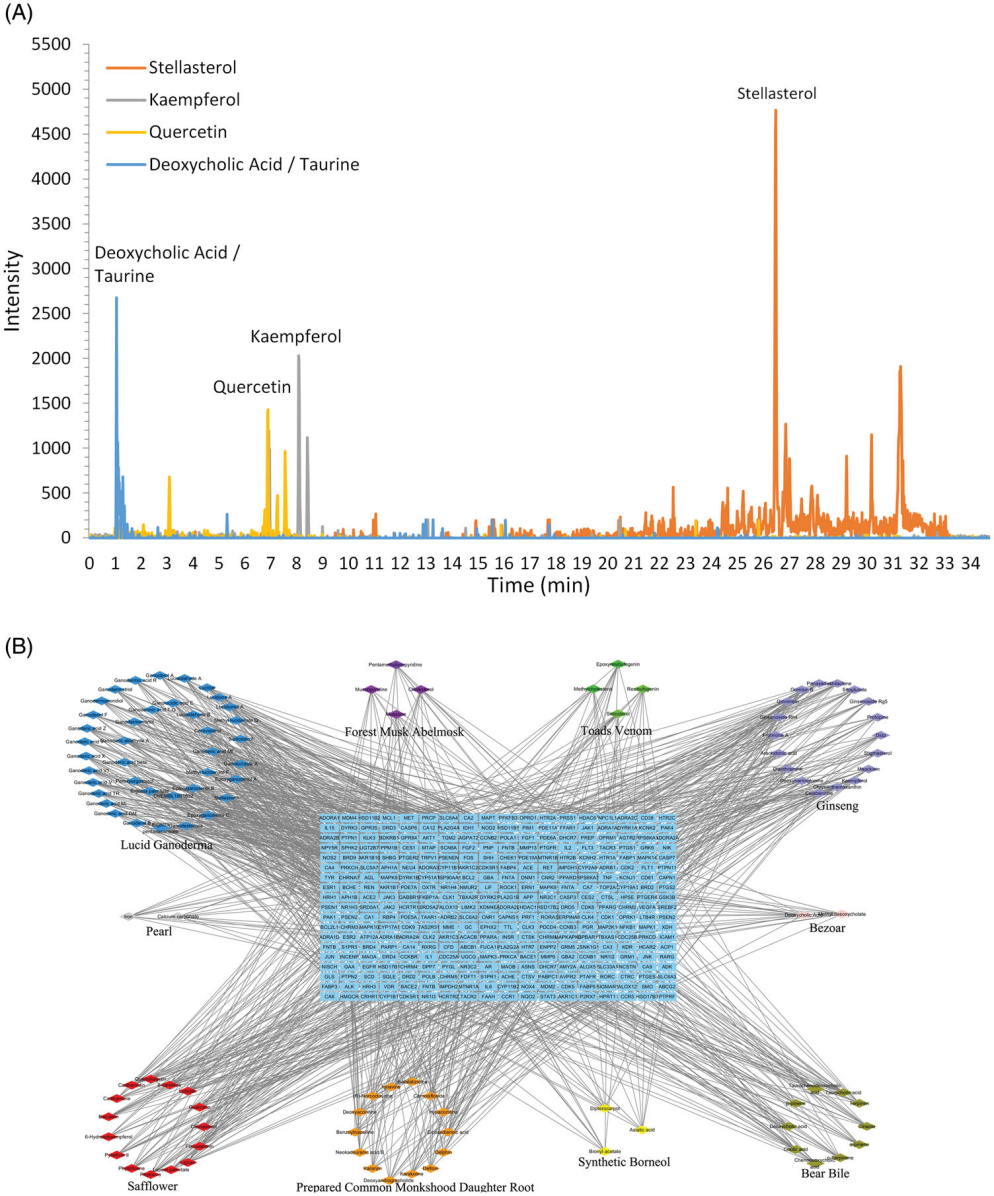
(A) HPLC profiles corresponding to stellasterol (orange), kaempferol (gray), quercetin (yellow), and deoxycholic acid/taurine conjugate (blue), shown according to their intensity peaks at different retention times. (B) Construction of the HXP compound-putative target network. The compound-putative target network was constructed by linking candidate compounds from the ten herbs, which are constituents of HXP, to their putative targets. The nodes representing candidate compounds are shown as polychromatic triangles, and the targets are indicated by blue squares.

We identified a total of 160 compounds contained within HXP, and among the top 20 compounds we identified a total of 16 bioactive compounds including (ginsenoside Ro, ginsenoside Rb1, gensenoside Rb2, lupenone, ginsenoside F4, ginsenoside Rd, echinocystic acid, licoricidin, ginsenoside Rg1, ginsenoside Rg3, daidzein, ginsenoside Rh3, ginsenoside Ra1, nicotiflorin, isoastragaloside I, and diosgenin) that were quantified using corresponding calibration curves of chemical standards and listed according to their corresponding intensities (Supplementary Figure S1). Moreover, we also identified HPLC profiles for the four high-degree components associated with multiple HXP targets from Tables 1 and 2 above, including stellasterol (main peak at 26.670 min), kaempferol (main peak at 8.053 min), quercetin (main peak at 6.788 min), and deoxycholic acid/taurine conjugate (main peak at 0.852 min), as shown in (Figure 2(a)).

### Identification of target proteins for HXP on myocardial infarction

**Figure 3.**
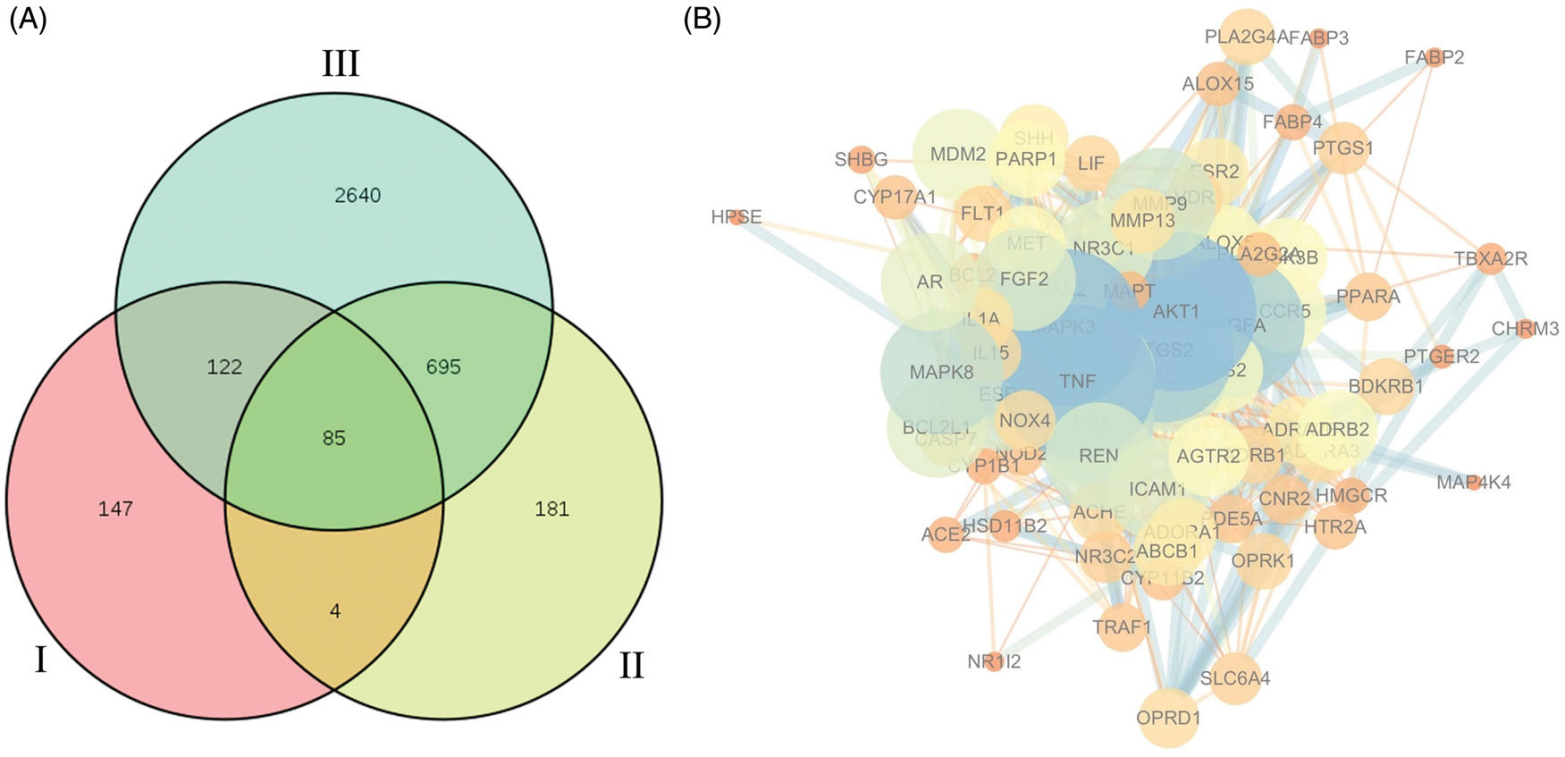
(A) Overlapping genes among 342 compounds-related genes (I), 281 MI-related genes from DisGeNet database (II) and 4643 MI-related genes from Genecards database (III). (B) A PPI network of candidate HXP targets for MI treatment extracted from the interactive PPI network of HXP putative targets and known MI-related targets.

Among the 111 candidate bioactive components of HXP, we retrieved a total of 1127 target proteins from the SwissTarget Prediction database. After eliminating overlapping proteins, we obtained a total of 342 associated target proteins. Next, we constructed a compound-protein network on the basis of the 111 bioactive compounds and their targets (Figure 2(b)), which was composed of 453 nodes (111 bioactive compounds and 342 targets). We further identified a total of 780 MI-related genes via data retrieval from the DisGeNET and Genecards databases. Venn diagrams showed that a total of 85 overlapping genes were identified by matching 90 compound-related genes with 780 MI-related genes (Figure 3(A)). Among these potential protein targets, there were six high-degree targets associated with multiple compounds, namely, AKT1 (degree = 53), VEGFA (degree = 45), TNF (degree = 51), and RELA (degree = 47) (Table 3). These high-degree protein targets in the network may account for the essential therapeutic effects of HXP on MI (Figure 3(B)).

**Table 3.** Targets of HXP on MI.

Number	Name	Degree	Betweenness Centrality	Number	Name	Degree	Betweenness Centrality
1	AKT1	53	0.107	41	ADORA3	13	0.003
2	VEGFA	45	0.053	42	NOX4	13	0.002
3	TNF	51	0.082	43	ACHE	13	0.006
4	PTGS2	46	0.089	44	FLT1	12	0.000
5	MAPK3	52	0.087	45	LIF	12	0.001
6	CASP3	40	0.022	46	PLA2G4A	12	0.001
7	MAPK1	38	0.020	47	OPRK1	12	0.002
8	STAT3	37	0.020	48	ADRB1	12	0.004
9	FOS	37	0.035	49	BDKRB1	12	0.011
10	MAPK8	37	0.017	50	VDR	12	0.001
11	ESR1	32	0.025	51	CASP7	11	0.000
12	MMP9	32	0.011	52	PTGS1	11	0.005
13	PPARG	30	0.051	53	NR3C2	11	0.003
14	ACE	30	0.035	54	SLC6A4	11	0.003
15	REN	30	0.029	55	TRAF1	10	0.000
16	FGF2	28	0.006	56	CNR2	10	0.002
17	ICAM1	28	0.008	57	PPARA	10	0.009
18	BCL2L1	27	0.005	58	PDE5A	10	0.001
19	NR3C1	26	0.026	59	BCL2	9	0.000
20	AR	26	0.015	60	CYP11B2	9	0.002
21	NFκB1	24	0.011	61	ALOX15	9	0.000
22	MDM2	23	0.003	62	CYP17A1	9	0.003
23	JAK2	22	0.006	63	HTR2A	9	0.016
24	NOS2	20	0.005	64	PLA2G2A	9	0.001
25	CCR5	19	0.011	65	NOD2	9	0.001
26	GSK3B	18	0.003	66	MAPT	8	0.000
27	AGTR2	18	0.011	67	FABP4	7	0.005
28	ADRB2	18	0.009	68	ACE2	7	0.001
29	PARP1	17	0.001	69	CYP1B1	7	0.003
30	MET	17	0.000	70	HMGCR	7	0.000
31	ALOX5	17	0.008	71	HSD11B2	6	0.000
32	ESR2	15	0.008	72	TBXA2R	6	0.008
33	ADRA2B	15	0.004	73	SHBG	5	0.000
34	ABCB1	15	0.008	74	PTGER2	4	0.001
35	ADORA1	15	0.008	75	CHRM3	3	0.000
36	SHH	15	0.001	76	FABP2	3	0.000
37	IL1A	14	0.004	77	FABP3	3	0.000
38	MMP13	14	0.000	78	NR1I2	3	0.000
39	OPRD1	13	0.003	79	MAP4K4	2	0.000
40	IL15	13	0.000	80	HPSE	2	0.000

### Molecular docking

**Figure 4.**
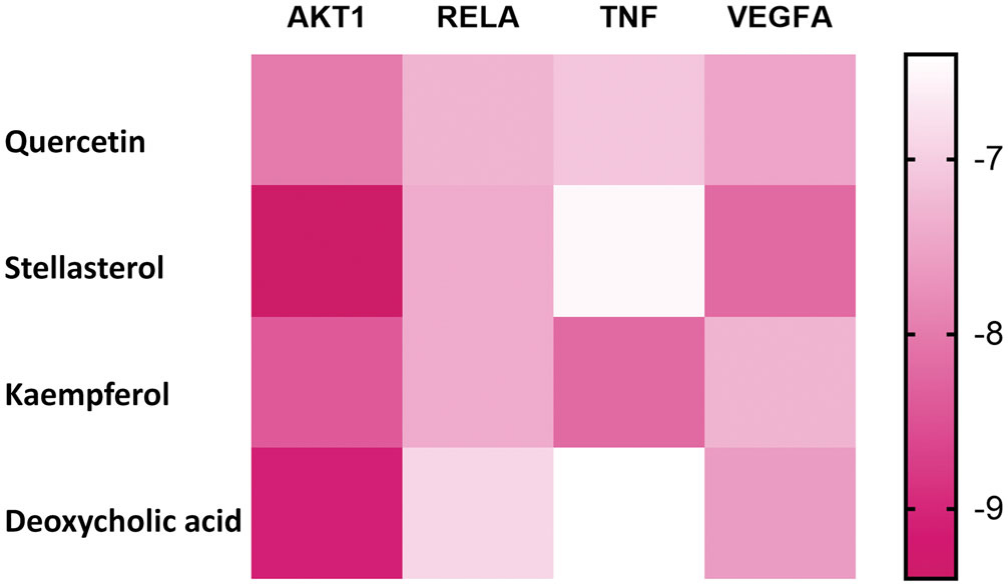
Molecular docking heat map. Molecular docking heat map indicating the scores of binding affinities that were highly binding (red) or lowly binding (white) following bioactive compounds and protein targets.

**Figure 5.**
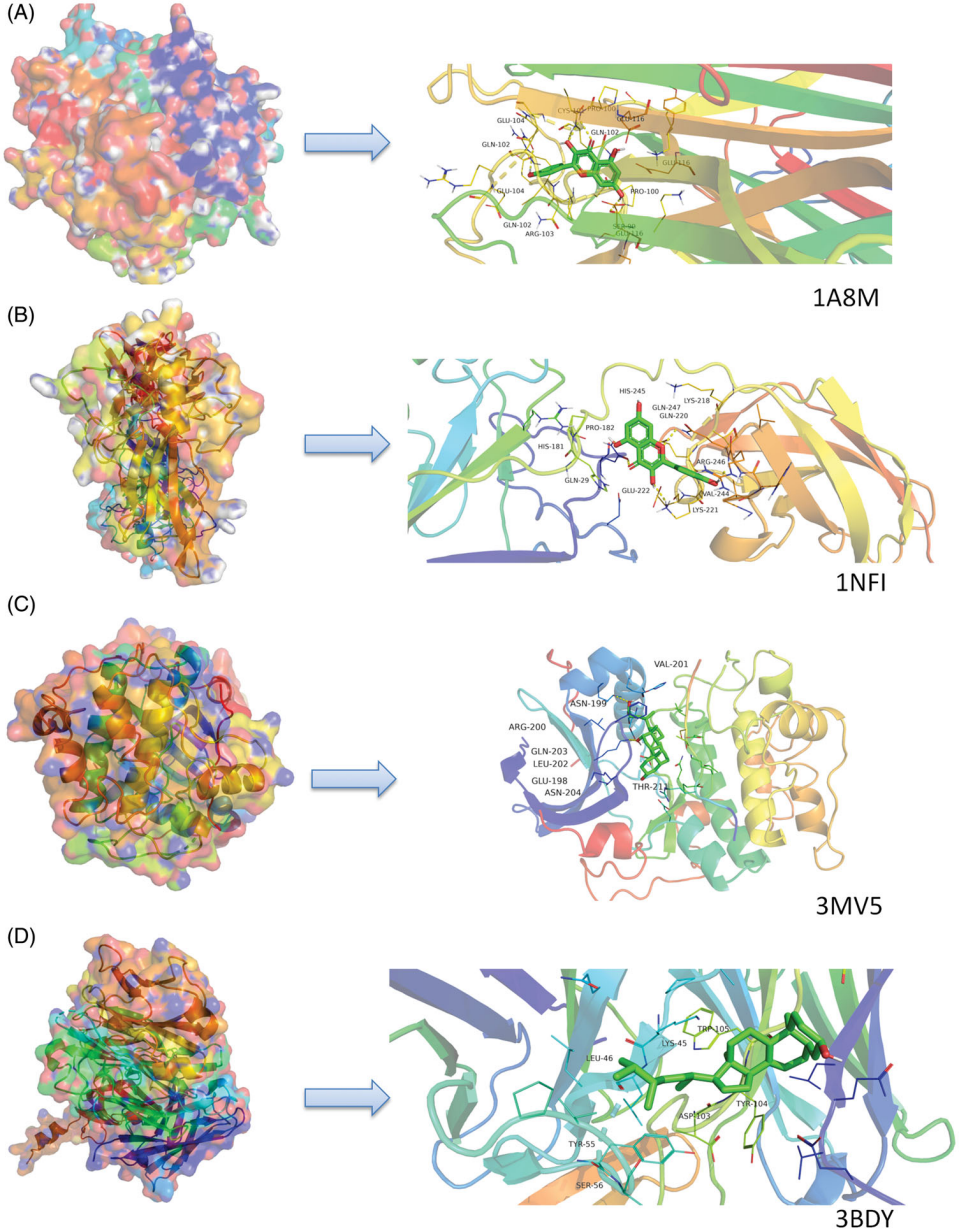
Molecular models of bioactive compounds that bind to their predicted protein targets. Green lines represent residues in the respective binding sites. Yellow dashed lines represent hydrogen bonds. Distance of interaction is indicated adjacent to the site of bonding. (A) 3D interaction diagrams of kaempferol in the active site of TNF (PDB ID 1A8M). (B) 3D interaction diagrams of stellasterol in the active site of RELA (PDB ID 1NFI). (C) 3D interaction diagrams of deoxycholic acid in the active site of AKT1 (PDB ID 3MV5). (D) 3D interaction diagrams of stellasterol in the active site of VEGFA (PDB ID 3BDY).

Molecular docking analysis provided a visual representation of the interactions between the major bioactive compounds in HXP with their potential protein targets associated with MI. In particular, kaempferol, stellasterol, deoxycholic acid, and quercetin all had binding energy of less than −6 kcal/mol with TNF, NFκB and TRAF1, demonstrating high binding affinities (Figure 4). Among all compounds, kaempferol possessed the highest binding affinity for TNF (Figure 5(A)), characterised by the formation of three hydrogen bonds. Stellasterol had the highest binding affinity for RELA, characterised by the formation of five hydrogen bonds in the active site of the target protein (Figure 5(B)). Deoxycholic acid and stellasterol showed the highest binding affinities towards Akt1 and VEGFA, respectively (Figure 5(C,D)).

### GO and pathway enrichment analysis

**Figure 6.**
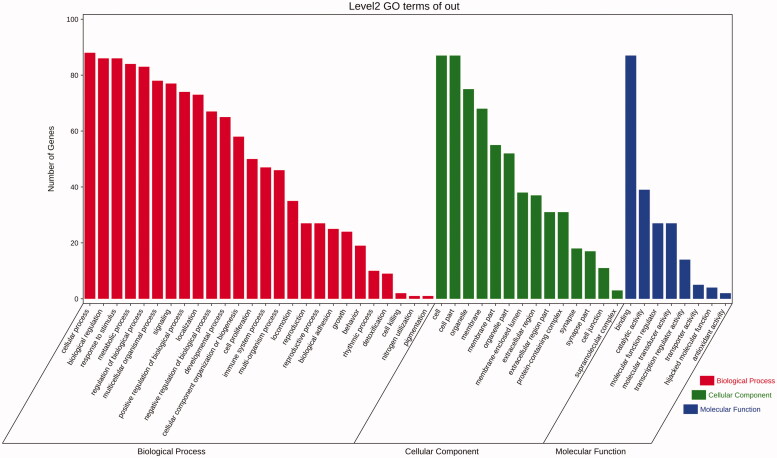
Gene ontology analysis of therapeutic target genes for HXP on MI.

**Figure 7.**
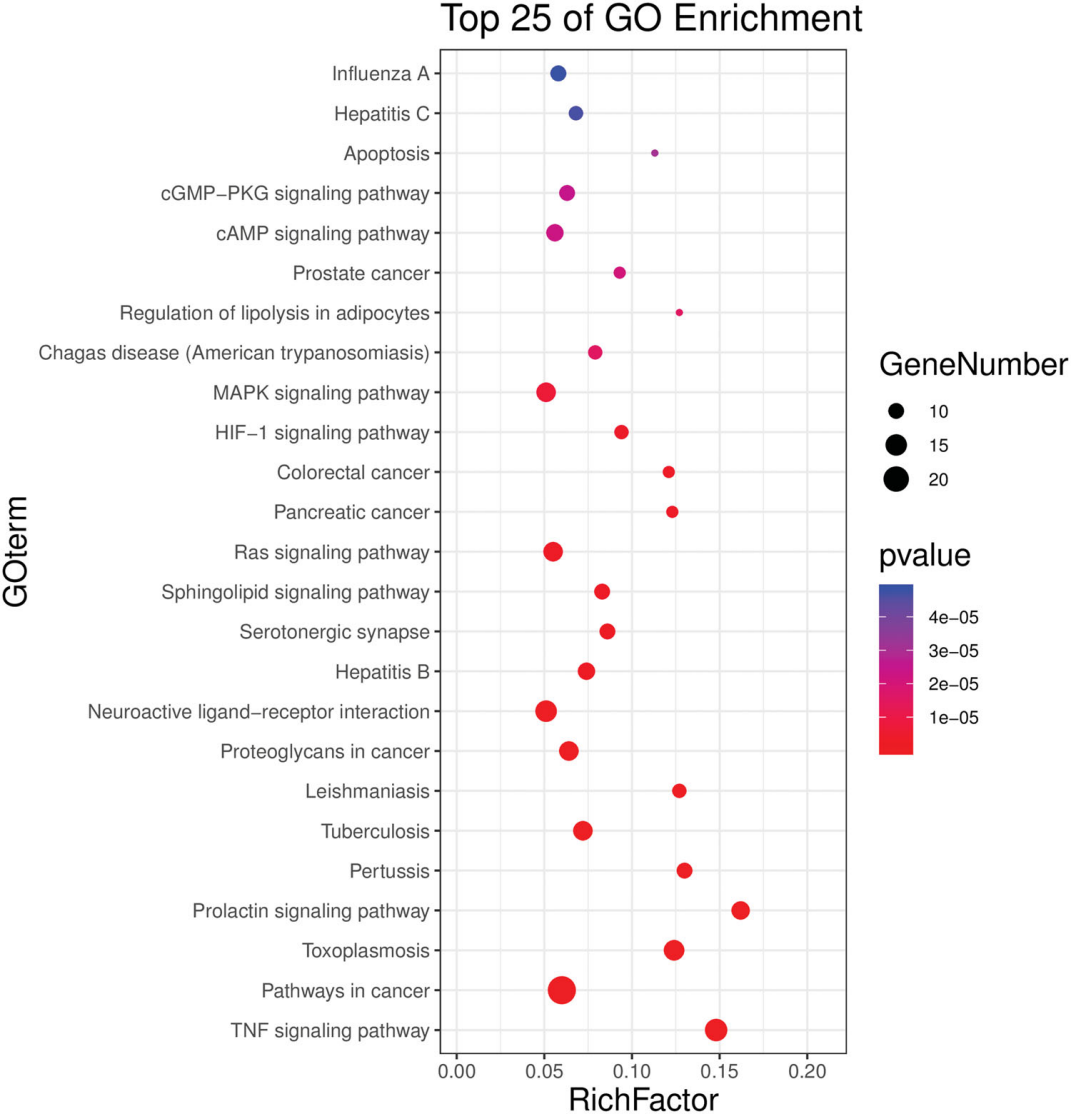
Pathway enrichment analysis of candidate targets for HXP in MI.

In order to analyse the biological characteristics of putative targets of HXP on MI in detail, we further conducted GO and pathway enrichment analyses of the target proteins using DAVID Bioinformatics Resources functional annotation tool. GO analysis showed that were a total of 148 biological processes (BP), 14 cellular components (CC), and 31 molecular functions (MF) associated with HXP on MI. Among these three categories, the top significantly enriched terms indicated that the therapeutic effects of HXP in protecting against MI may involve the regulation of cell proliferation via molecular transducer activity, molecular function regulator, and catalytic activity in synapse, membrane, and extracellular regions (Figure 6). In order to better understand the underlying mechanisms involved in the cardioprotective effects of HXP on MI, we conducted KEGG pathway analysis of these target genes. Of the top 25 significantly enriched pathways of HXP on MI, TNF pathway had the highest significance and degree value (Figure 7). Among its potential targets, TNF, TRAF1, and NFκB were identified as relatively high-degree targets that play an essential role in the inflammatory response, and thus are the potentially key genes involved in the cardioprotective effects of HXP on MI. Taken together, we speculate that the therapeutic effects of HXP on MI are likely via the improved regulation of the inflammatory response.

### HXP improves cardiac function following MI *in vivo*

**Figure 8.**
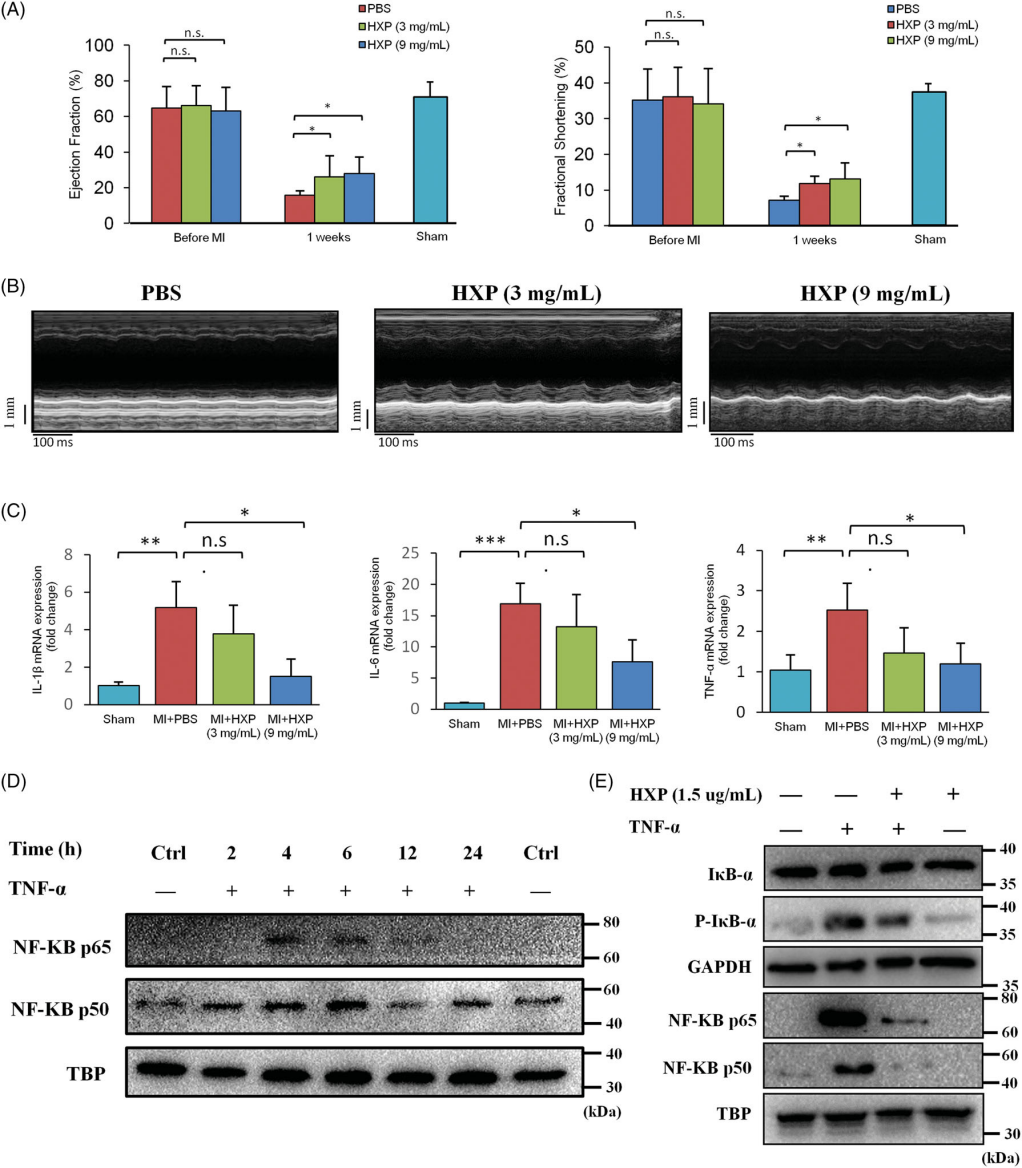
HXP prevents excessive inflammation after myocardial infarction. (A) Echocardiography parameters: ejection fraction (EF%), and fractional shortening (FS%), measured at 1 week post-MI in PBS-treated and HXP-treated (3 and 9 mg/mL) mice. *p < 0.05. **p < 0.01. *n* = 10 for each. (B) Representative M-mode echocardiography at 1 week post-MI showing LV end-systolic and end-diastolic dimensions in PBS-treated and HXP-treated (3 and 9 mg/mL) mice. (C) Real-time PCR results showing the relative expressions of pro-inflammatory cytokines IL-1β, IL-6 and TNF-α in the infarct regions of PBS-treated and HXP-treated (3 and 9 mg/mL) mice at 1 week following MI. Error bars represent SEM of the fold changes in upper ΔCT values after normalisation with GAPDH. *p < 0.05. **p < 0.01. (D) Western blots showing the nuclear expression of NF-κB following TNF-α treatment at varying times in adult H9c2 cardiomyocytes. TBP, loading control. (E) Western blots showing the expressions of Iκb-α, p-Iκb-α and nuclear NF-κB following pre-treatment with HXP for 12 h and subsequent TNF-α treatment for 4 h in adult H9c2 cardiomyocytes. GAPDH: total loading control; TBP: nuclear loading control.

We further performed experimental validation of our pharmacology based analysis. To verify the cardioprotective effects of HXP following MI, we performed mouse models of MI and administered PBS or HXP (3 or 9 mg/mL) following MI via oral gavage. No differences in mortality rates (≈10%) were observed at 1 week post-MI. Echocardiography results showed that at 1 week post-MI, mice treated with HXP (both 3 and 9 mg/mL) had significantly improved LV wall motion along with higher ejection fraction (EF) and fractional shortening (FS) parameters compared to mice treated with PBS (Figure 8(A,B)). These results demonstrated the robust cardioprotective effect of HXP following MI, which was most prominent at higher dose (9 mg/mL).

### HXP reduces inflammatory response following MI *in vivo*

In order to verify that the action of HXP in protecting against MI was via regulating the inflammatory response, we examined the expression of inflammatory cytokines using real-time PCR analysis. At 1 week post-MI, the expression levels of pro-inflammatory cytokines IL-1β, IL-6 and TNF-α were significantly upregulated compared to Sham-operated mice (Figure 8(C)). Interestingly, although mice treated with lower dose HXP (3 mg/mL) had slight reductions in expression of inflammatory cytokines, those treated with higher dose HXP (9 mg/mL) had significantly decreased expression levels of IL-1β, IL-6, and TNF-α following MI compared to PBS-treated mice. These results suggested that HXP improved cardiac ischaemic injury via reducing the inflammatory response, and that the pharmacodynamics of HXP required the use of a higher dose (9 mg/mL) for the most effective inhibition of adverse inflammatory response following MI.

### HXP prevents TNF-Induced inflammation* in vitro*

We next examined the effect of HXP in reducing inflammation response *in vitro* using adult rat myoblast H9c2 cells. Cells were treated with TNF-α at various time intervals, and the inflammatory response was shown to be significantly activated at 4 h following treatment, as indicated by the increase in nuclear expression of NF-κB heterodimers p50 and p65 (Figure 8(D)). Furthermore, pre-treatment with HXP significantly decreased the levels of inflammatory cytokines IκB-α, and phospho-IκB expression following TNF-α treatment (Figure 8(E)). Notably, HXP pre-treatment also significantly attenuated TNF-α-induced increase in nuclear NF-κB expression. These results indicated that the protective action of HXP was via alleviating the inflammatory response.

## Discussion

In the event of acute MI, the sudden death of cardiomyocytes in the infarcted heart rapidly stimulates the activation of innate immune pathways, which in turn activates an intense inflammatory response that clears the infarcted area of dead cells and extracellular matrix debris (Frangogiannis 2008). However, prolonged or excessive inflammation is associated with worsened cardiac injury and adverse cardiac remodelling (Saparov et al. 2017). Therefore, therapeutics that can control or limit the inflammatory response following MI is a promising form of treatment.

TCM is composed of multiple compounds that may exhibit positive pharmacological activities in the treatment of MI. Using a network pharmacological analysis, we systematically investigated the drug targets, associated pathways and networks involved in the mechanism of HXP in protecting against MI. In the compound-target network, high-degree targets may likely account for the main therapeutic effects of HXP. Thus, stellasterol was considered the most important active ingredient of HXP, which had been demonstrated to have various anti-cardiovascular and anti-inflammatory properties (Seo et al. 2009). Deoxycholic acid is known to induce apoptotic resistance via NF-κB activation (Huo et al. 2011), while both kaempferol and quercetin are dietary flavonoids that are associated with a lower incidence of coronary heart disease. Kaempferol can significantly decrease the level of inflammatory markers, including TNF and NF-κB (Yoon et al. 2013; Al-Numair et al. 2015), while quercetin exhibits anti-inflammatory and anti-apoptotic effects on coronary heart disease (Li et al. 2016). Thus, these compounds likely play key roles in the therapeutic effect of HXP in protecting against MI.

Our analysis showed that the highest correlated targets between MI-related genes and HXP compound-related genes were AKT1, TNF, NFκB, PTGS2 and VEGFA, which are mainly involved in the processes of angiogenesis and inflammation. AKT plays an essential role in VEGF-mediated angiogenesis following cardiac ischaemia, where increased endothelial cell migration can slow down the rate of cardiac dysfunction (Rotllan et al. 2015). In addition, the active HXP-derived compounds β-pinene, dianthramine, arachidonic acid, eicosadienoic acid and carthamidin can inhibit Prostaglandin G/H synthases PTGS1 and PTGS2 that are involved in the conversion of arachidonate to prostaglandins, which are critical cardiovascular system mediators of inflammatory processes (Agundez et al. 2014). NF-κB is a pleiotropic transcription factor present in almost all cell types and is the endpoint of a series of signal transduction events that are initiated by a vast array of stimuli related to many biological processes of inflammatory processes (Chen et al. 2002).

By predicting and analysing putative targets, we speculated that the therapeutic effect of HXP in protecting against MI is likely via improving the inflammatory response. TNF-α is actively involved in triggering inflammation and cardiac wound healing processes following MI. The biological activity of TNF-α is mediated via two distinct cell-surface receptors, TNFR-1 that initiates the caspase cascade triggering apoptosis, and TNFR2 that initiates cell survival and angiogenesis via NF-κB signalling pathway (Higuchi et al. 2006). NF-κB plays a major role in TNF-α-mediated cardiotoxicity, and its molecular structure contains an inhibitory κB protein (IκB), which acts to inhibit NF-κB function (Baldwin 1996). NF-κB signal transduction pathway plays key roles in regulating immunity, inflammation and cell survival. Our experimental validation using TNF-α treatment in H9c2 adult cardiomyocytes demonstrated that TNF-α inactivated IκB via the phosphorylation of serine/tyrosine residues, resulting in IκB degradation/dissociation from NF-κB. This activation of the inflammatory response led to the rapid expression of a wide variety of pro-inflammatory cytokines, chemokines and their receptors, including IL-1β, IL-6 and IL-8 (McDermott and O’Neill 2002). These pro-inflammatory cytokines further promote inflammatory cell adhesion and infiltration into the myocardium, causing obstruction of capillary vessels, production of vasoactive substances, and release of cytotoxic agents. In addition, *in vivo* experiments verified our network pharmacology analysis results, which showed that the cardioprotective effect of HXP following myocardial ischaemia was likely via improving the regulation of the inflammatory response. Adverse inflammatory stimuli following MI can exacerbate the degree of ischaemic injury and delay the recovery of cardiac function (Kain et al. 2014). However, further studies are necessary to investigate the detailed mechanisms by which HXP ameliorates the inflammatory response following MI. Our study thus provides scientific evidence to support the therapeutic effect of HXP and the basis for further research into the active ingredients and compounds contained in HXP in protecting against cardiac ischaemic injury.

## Conclusions

Our study utilised a network pharmacology based analysis to select for the main active compounds and target genes involved in the therapeutic effect of HXP. We demonstrated that the action of HXP in protecting against MI was mainly via the improved regulation of the inflammatory response, which provides a theoretical basis for the clinical application of HXP in treating patients with angina or myocardial ischaemia. Future research into the combination of surgical procedures or medications that restore blood flow together with HXP as supportive medication would be promising.

## Supplementary Material

Supplemental Material

Supplemental Material

## Data Availability

The data used and analysed during the current study are available from the corresponding author on reasonable request.
