# Supplementary figures and images for: Network pharmacology-based analysis in determining the mechanisms of Huoxin pill in protecting against myocardial infarction
Source: Pharm Biol. 2021 Sep 7;59(1):1189–200. doi: 10.1080/13880209.2021.1964542 (PMC8425702; doi:10.1080/13880209.2021.1964542)

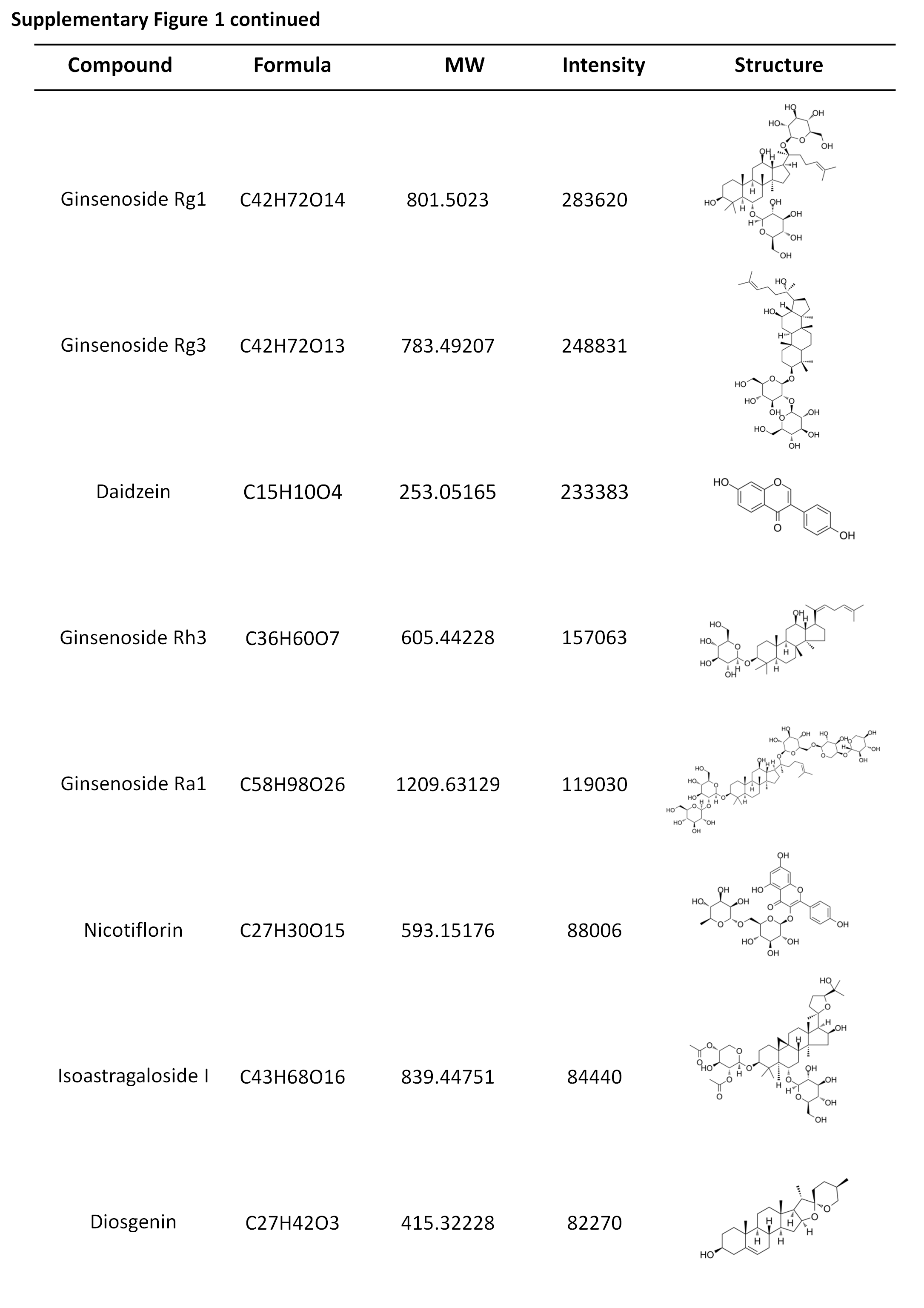

Supplement: Supplemental Material [file IPHB_A_1964542_SM6761.docx]

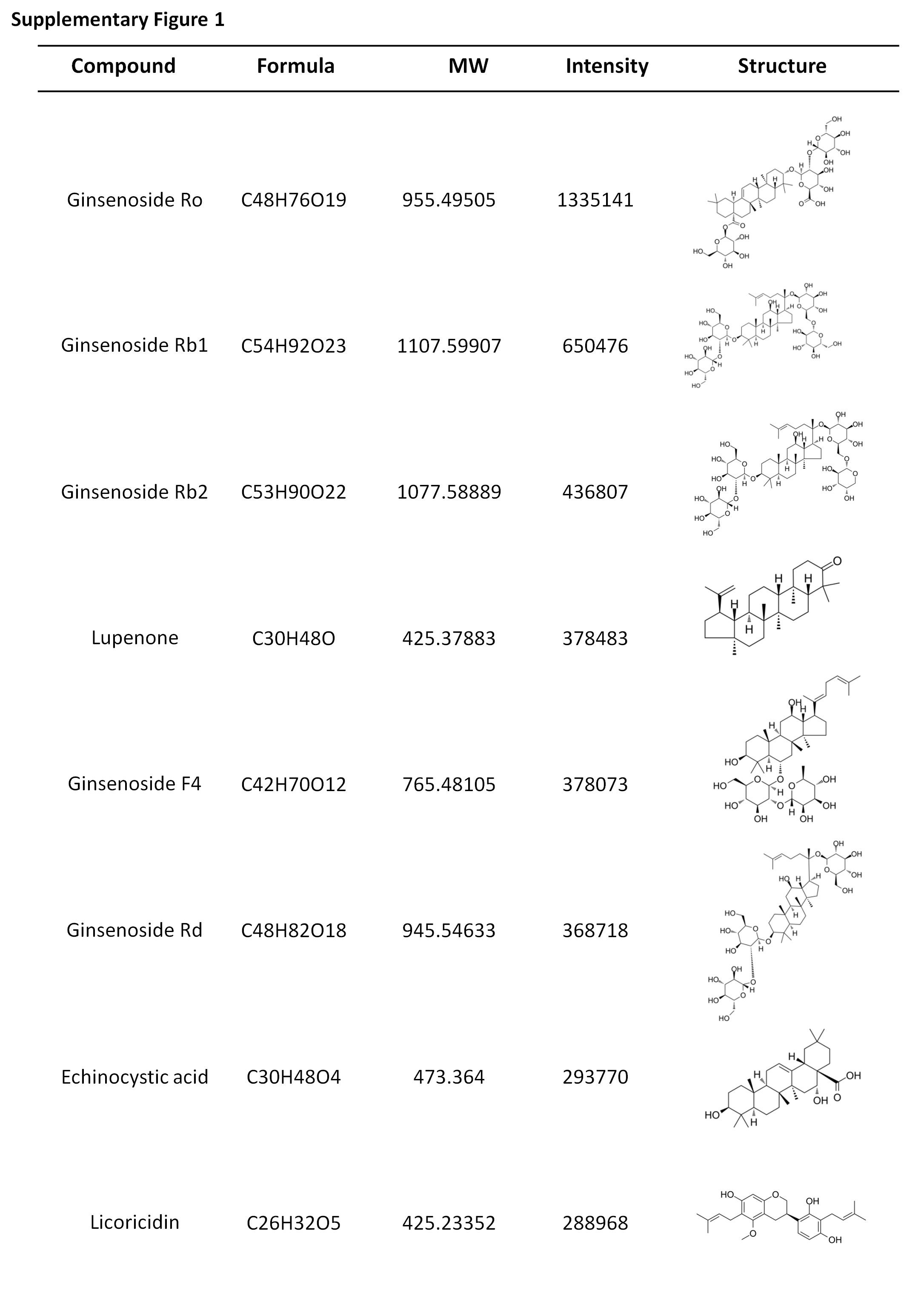

Supplement: Supplemental Material [file IPHB_A_1964542_SM6758.docx]
